# Functional Analysis of Kinases and Transcription Factors in *Saccharomyces cerevisiae* Using an Integrated Overexpression Library

**DOI:** 10.1534/g3.116.038471

**Published:** 2017-01-22

**Authors:** Ji-Young Youn, Helena Friesen, Alex N. Nguyen Ba, Wendy Liang, Vincent Messier, Mike J. Cox, Alan M. Moses, Brenda Andrews

**Affiliations:** * Department of Molecular Genetics, University of Toronto, Ontario M5S 3E1, Canada; † Terrence Donnelly Centre for Cellular and Biomolecular Research, University of Toronto, Ontario M5S 3E1, Canada; ‡ Department of Cell and Systems Biology, University of Toronto, Ontario M5S 3B2, Canada; § Centre for the Analysis of Genome Evolution and Function, University of Toronto, Ontario M5S 3B2, Canada

**Keywords:** yeast genetics, genetic interactions, kinase, transcription factor, genetic networks

## Abstract

Kinases and transcription factors (TFs) are key modulators of important signaling pathways and their activities underlie the proper function of many basic cellular processes such as cell division, differentiation, and development. Changes in kinase and TF dosage are often associated with disease, yet a systematic assessment of the cellular phenotypes caused by the combined perturbation of kinases and TFs has not been undertaken. We used a reverse-genetics approach to study the phenotypic consequences of kinase and TF overexpression (OE) in the budding yeast, *Saccharomyces cerevisiae*. We constructed a collection of strains expressing stably integrated inducible alleles of kinases and TFs and used a variety of assays to characterize the phenotypes caused by TF and kinase OE. We used the Synthetic Genetic Array (SGA) method to examine dosage-dependent genetic interactions (GIs) between 239 gain-of-function (OE) alleles of TFs and six loss-of-function (LOF) and seven OE kinase alleles, the former identifying Synthetic Dosage Lethal (SDL) interactions and the latter testing a GI we call Double Dosage Lethality (DDL). We identified and confirmed 94 GIs between 65 OE alleles of TFs and 9 kinase alleles. Follow-up experiments validated regulatory relationships between genetically interacting pairs (Cdc28–Stb1 and Pho85–Pdr1), suggesting that GI studies involving OE alleles of regulatory proteins will be a rich source of new functional information.

Protein phosphorylation modulates many cellular activities in eukaryotes, and hyperactivation of kinases and their transcriptional targets is often associated with oncogenesis and other disease phenotypes (Blume-Jensen and Hunter 2001). In yeast, at least 73% of TFs are phosphorylated *in vivo* (Stark *et al.* 2010); however, less than half of these phosphorylation events have been associated with a cognate kinase (Bodenmiller *et al.* 2010; Stark *et al.* 2010; Sharifpoor *et al.* 2011). Identifying direct kinase–TF relationships remains difficult due to the pleiotropic function of kinases and TFs *in vivo* and the large spectrum of potential interactions revealed by *in vitro* studies (Ubersax *et al.* 2003; Ptacek *et al.* 2005), requiring multiple levels of functional assays to support their *bona fide* relationship.

One approach to interrogate kinase–TF regulatory relationships is to examine GIs involving kinase and TF mutants. In yeast, SGA technology has been used to systematically survey GIs between LOF alleles of nonessential genes, and between temperature-sensitive alleles of essential genes, and the resultant GI networks have proven to be rich in functional information (Tong *et al.* 2001; Costanzo *et al.* 2010, 2016). GIs occur when a combination of perturbations, in two or more genes, generates a phenotype deviating from the phenotype expected based on the single mutant phenotypes (Costanzo *et al.* 2010). A negative GI occurs when the double mutant displays a more severe phenotype than expected, such as cell death (synthetic lethality). Conversely, a positive GI occurs when the double mutant phenotype is less severe than expected. GIs involving LOF alleles of kinases and phosphatases have been specifically assessed, revealing functional redundancies and regulatory relationships (Fiedler *et al.* 2009; van Wageningen *et al.* 2010). SGA analysis can also be used to analyze GIs involving other types of genetic perturbations, including gene OE. SDL occurs when increased dosage of one gene exacerbates the phenotype caused by a LOF mutation in another gene, resulting in an extreme slow growth phenotype or lethality (Kroll *et al.* 1996; Measday and Hieter 2002; Sopko *et al.* 2006; Kaluarachchi Duffy *et al.* 2012; Sharifpoor *et al.* 2012). SDL screens have proven fruitful for analyzing enzyme–substrate relationships and the integration of different SL and SDL interactions has enabled discovery of network motifs that are highly predictive of functional relationships between kinases and their targets (Sharifpoor *et al.* 2012).

Several mechanisms have been proposed to explain dosage toxicity. First, increased gene dosage may lead to protein hyperactivity, as increased protein levels may prevent appropriate regulation (hypermorph; Prelich 2012). Alternatively, an overproduced protein may phenocopy a LOF phenotype (hypomorph), if an imbalance in the concentration of protein complex components impairs the function of the complex (the “balance hypothesis;” Papp *et al.* 2003). A third mechanism of OE toxicity is suggested by the observation that dosage toxicity of a protein is highly correlated to its intrinsic disorder content (Vavouri *et al.* 2009). Intrinsically disordered regions are involved in protein–protein interactions (PPIs) (Dunker *et al.* 2001), protein degradation (Brocca *et al.* 2009), and post-translational modifications (Dunker *et al.* 2002), and these functions are mediated by short (2–10 aa long) linear motifs that are often conserved (Obenauer *et al.* 2003; Nguyen Ba *et al.* 2012). Because linear motifs are short, degenerate, and bind target proteins with low affinity, increased dosage may lead to off-target binding events, creating toxic interactions by mass action (Vavouri *et al.* 2009). This so-called interaction promiscuity hypothesis predicts that protein OE will lead to neomorphic phenotypes distinct from those associated with the wild-type protein.

In order to explore mechanisms of dosage toxicity and their utility for mapping biological pathways, we focused on kinases and TFs, which often have a regulatory relationship that is readily perturbed by dosage (Chua *et al.* 2006; Sopko *et al.* 2006). To enable systematic genetic analysis, we constructed SGA-compatible libraries of strains carrying wild-type alleles of kinases or TFs that can be conditionally overproduced by induction of the *GAL* promoter, all integrated at the benign *ho* locus. We performed a phenotypic analysis of cell morphology and growth in each OE strain, and examined GIs between LOF alleles of kinases and OE alleles of TFs (SDL). We confirmed 68 interactions between 4 kinase alleles and 52 TF OE alleles. The SDL interactions identified using analog-sensitive (*as*) alleles of *CDC28* and *PHO85* (*cdc28-as1* and *pho85-as*) were enriched for known enzyme–substrate pairs, including the TF Stb1, whose nuclear localization was regulated by Cdc28. Finally, we explored a new GI, which we dubbed DDL, using a subset of kinase alleles. DDL describes a GI that occurs when the increased level of a protein has little impact on its own but results in a phenotype that is more severe than expected, such as lethality, when combined with overproduction of a second protein. Our DDL screens identified a network of 26 confirmed interactions between 5 cyclin OE alleles and 25 TF OE alleles. These interactions included several previously characterized kinase–substrate pairs as well as a novel kinase–substrate relationship between the Pho85-Pho80 CDK complex and a TF involved in the pleiotropic drug response, Pdr1.

## Materials and Methods

### Strain construction, growth conditions, and determination of fitness

To build constructs containing 432 different ORF sequences (149 kinases and 283 TFs), we used Gateway recombination technology to transfer the majority of these ORFs from the movable ORF (MORF) collection (Gelperin *et al.* 2005; Supplemental Material, Table S1 and Table S2 in File S1). For TFs and kinases missing from the collection, and those for which the ORF was inconsistent with the predicted size in the MORF collection, we used ORFs from the FLEX collection (Hu *et al.* 2007) or from a Flag-tagged library (Breitkreutz *et al.* 2010). We moved each ORF fragment from the relevant library into a Gateway ENTRY vector, then into a Gateway-compatible destination expression vector that we designed to target integration at the benign *ho* locus. The *ho*-targeting vectors were derived from *HO*-poly-KanMX4-*HO* (Voth *et al.* 2001) and were designed for N- or C-terminal Flag-tagged expression of the ORF (BA433V and BA2262, respectively) under the control of the *GAL* promoter, marked by a NAT-MX resistance cassette. The source of the ORF sequence determined the N- or C-terminal tagging (for instance, ORFs from the MORF collection are C-terminally tagged, whereas those from the FLEX collection are N-terminally tagged; indicated in Table S1 and Table S2 in File S1). The integration vectors contained sequences homologous to the promoter and 3′-UTR regions of the *ho* gene, allowing the entire construct (*NATMX*::*GAL promoter-ORF-FLAG*::*CYC1 terminator*) to replace the *ho* locus. All integration constructs were transformed into strain Y7092, the SGA *MAT*α query strain (Tong and Boone 2007; see Table S3 in File S1 for details). Quality control steps included: (i) verification of the ORF size at every Gateway step by restriction digest; (ii) checking proper integration of the construct at the *ho* locus by PCR; and (iii) confirmation of galactose-induced protein expression following the induction procedure described below, by western blot analysis.

Standard methods and media were used for yeast transformation and growth (Guthrie and Fink 1991). For induction of protein OE, each OE strain was grown to saturation overnight in rich medium containing 2% raffinose (YPRaf), then diluted to an OD_600_ of 0.1–0.2 in rich medium containing 2% galactose (YPGal) to induce gene OE and incubated for 4–5 hr. For western blot analysis, cells were grown in 1.2 ml volumes in 96-well blocks, collected after 4–5 hr induction, washed, and frozen for lysate preparation using trichloracetic acid extraction (Kurat *et al.* 2009). Monoclonal α-FLAG antibody (Sigma, F3165) was used for detection of galactose-induced protein. Quantification of protein levels was done using Quantity One software.

For fitness assessment, 2 μl of saturated cultures was transferred to 98 μl of YPGal to achieve an OD_600_ of ∼0.1. Cultures were grown in biological triplicate in 96-well plates in Tecan GENios microplate readers for 24 hr, with OD measurements taken every 15 min. The doubling time was determined by calculating the difference between the time after five doublings (*t*_5_) and time after two doublings (*t*_2_) and dividing this by three [*D =* (*t*_5_ − *t*_2_)/3], as described (St Onge *et al.* 2007). Fitness was normalized to wild-type; OE fitness = *D*_wild-type_/*D*_OE_.

To synchronize the Stb1-GFP strain from GFP collection, α-factor block and release was performed (Huh *et al.* 2003). Cells were grown to early log phase (OD_600_ of ∼0.2) in YPD, then arrested in G1 phase with 5 μM α-factor (GenScript) for 2.5 hr at 30°. Cells were washed twice in 0.5 volumes of YPD, released into YPD, and samples were collected every 15 min for western blotting.

### Microscopy for morphological profiling and analysis of GFP-tagged TF localization

For live-cell imaging, cells were grown at 30° in 96-well blocks with beads and shaken at 200 rpm to maintain the cells in suspension. TF or kinase OE was induced as described above. Cells were transferred to a 96-well imaging slide (Matrical, MGB096-1-2-LG), briefly centrifuged (1000 rpm for 30 sec), and imaged at room temperature using a DMI 6000B fluorescence microscope (Leica Microsystems) equipped with a spinning-disk head, an argon laser (458, 488, and 514 nm; Quorum Technologies, Guelph, ON, Canada), and an ImagEM-charge-coupled device camera (Hamamatsu C9100-13, Hamamatsu Photonics, Hamamatsu City, Japan). 16-bit images were analyzed using Volocity software (Improvision, Coventry, United Kingdom). Images were taken using DIC, and in the GFP and RFP channels, and cell morphological defects were qualitatively scored by eye using a selected list of seven categories and 17 subcategories (Table S4 in File S1).

Twenty-one C-terminally-tagged TF-GFP strains (Huh *et al.* 2003) were crossed to *ura3*Δ (BY4065) and *cdc28-as1* (BY4055) strains (Table S3 in File S1) to generate WT *TF-GFP* and *cdc28-as1* TF-GFP strains using the SGA method (Tong *et al.* 2001). Saturated cultures were diluted in SC medium and grown for 4–5 hr to log phase, then incubated in a 30° shaker, with either carrier (DMSO) or 5 μM 1-NM-PP1 for 40–50 min. Cells were then transferred to a 96-well imaging slide and imaged using DIC and in the GFP channel at room temperature using a confocal microscope (see above).

### SGA analysis and confirmation using serial spot dilutions

Strains carrying integrated OE alleles of TFs (239 TFs) were pinned in quadruplicate to create one 1536-format array. Mutant alleles of kinases (Giaever *et al.* 2002) and wild-type control (*ura3*Δ::*KAN* in BY4741 background; Brachmann *et al.* 1998) strains were introduced into the array using SGA technology (see Table S3 in File S1 for strain list; Tong *et al.* 2001). The resultant haploid mutants carrying OE alleles were pinned onto galactose-containing medium and colony size was measured to determine double mutant fitness (Tong and Boone 2006). For *cdc28-as1* and *pho85-as* screens, 3 μM 1-NM-PP1 and 50 μM 1-Na-PP1, respectively, was added to the final selection plates. Since the scale of these screens was relatively small, we first identified GIs by eye and then confirmed the interactions by manually regenerating the double mutant strains using SGA haploid selection markers and testing the fitness of the double mutant strains compared to the relevant single mutants by using a spot dilution assay. For spot assays, overnight cultures grown in raffinose-containing synthetic media were diluted to OD_600_ of 1–2, then serially diluted 15-fold five times and spotted onto both noninducing control SD plates and inducing SGal plates. To confirm *cdc28-as1* and *pho85-as* interactions, we used SG plates with 50 nM 1-NM-PP1 and 0.9 μM 1-Na-PP1, respectively, determined after testing different concentrations of inhibitors for best spot assay resolution. SD and SGal plates were grown for 2 and 3 d at 30°, at which point viability of each single and double mutant was scored by counting the number of spots. The number of spots was used to calculate the strength of each GI in a semiquantitative manner; we subtracted the number of expected viable spots in the double mutant from the number observed to estimate the deviation of double mutant fitness from the expected value (GI score = observed fitness − expected fitness). For instance, in spot dilution experiments where a wild-type strain shows growth of five spots, if a kinase mutant alone has five viable spots (fitness 5/5 = 1) and a TF OE strain alone has four out of five viable spots (fitness 4/5 = 0.8), we predict that, if there is no GI, the double mutant fitness will be the product of the two single mutant fitnesses (fitness = 0.8). A GI occurs when the double mutant shows fewer or more than four viable spots. In the example described above, if the double mutant shows one out of five viable spots (fitness 1/5 = 0.2), a GI score can be calculated by subtracting the expected double mutant fitness from the observed fitness (GI score = 0.2 − 0.8 = −0.6). This score is then used as a semiquantitative measure of GI strength. In Table S9 and S10, the GI score was multiplied by 5 to derive ‘Genetic Interaction Strength’ value, ranging from -5 to 5.

### Analysis of protein toxicity and correlated features

The length of disordered regions in each protein was estimated using the DISOPRED2 database (Ward *et al.* 2004). Conserved linear motifs within disordered regions were identified as previously described (Nguyen Ba *et al.* 2012). When assessing the number of PPIs associated with each kinase and TF, we extracted binary and complex-associated PPI information using only data from high-throughput studies to exclude biases in the literature often found with well-characterized proteins (Sharifpoor *et al.* 2011). We also excluded those with no annotated physical interaction to eliminate those that may have never been tested.

### Antibodies and western blots

Western blot analysis was performed using standard procedures. For Phos-tag gels, we followed the instructions provided in the manual. Antibodies used in this study were anti-Clb2 (Santa Cruz, sc-9071), monoclonal anti-GFP (Living Colors), anti-Hexokinase (Rockland, Gibertsville, PA), and monoclonal anti-FLAG antibody (Sigma, F3165). To measure the relative levels of the overexpressed FLAG-tagged proteins, we compared the intensity of the FLAG-tagged protein band to that of a 100 kDa protein band, which is nonspecifically detected by the anti-FLAG antibody and is present in consistent amounts in all protein extracts tested.

### Protein-fragments complementation assay (PCA)

We performed PCA using strains in which each protein was fused to one of the complementary dihydrofolate reductase (DHFR) PCA fragments (Tarassov *et al.* 2008). As a measure of physical interaction, we used spot dilutions to detect association of test proteins fused to DHFR fragments. All strains were grown overnight in 5 ml of SD medium supplemented with antibiotics. Cultures were adjusted to an OD_600_ of 1, serially diluted 15-fold, and 5 μl of each dilution was spotted on methotrexate-containing medium (200 μg/ml, Bioshop). Plates were incubated for 6 d at 30° and imaged.

Strains are available upon request. Table S1, Table S2, and Table S3 in File S1 contain detailed descriptions of all strains generated in this study. Table S5, Table S6, and Table S8 in File S1 provide further details on fitness and morphology defects associated with each strain.

### Data availability

The authors state that all data necessary for confirming the conclusions presented in the article are represented fully within the article.

## Results

### Construction of kinase and TF OE libraries

In our experiments using OE genetics to explore the relationship between protein kinases and TFs, we defined the kinase gene set for budding yeast as the 127 proteins with a predicted kinase domain (Rubenstein and Schmidt 2007), and included 22 cyclins that are regulatory subunits of cyclin-dependent kinases (CDKs) (see Table S1 in File S1). To define a TF set, we relied on previously defined TFs in the YeTFaSCo (Yeast Transcription Factor Specificity Compendium) database (de Boer and Hughes 2012), which includes any yeast protein that contains an annotated DNA-binding domain or that shows DNA binding to a characterized DNA sequence. We also included proteins that were both defined as TFs and tested for *in vitro* DNA-binding specificity (Badis *et al.* 2008), as well as 23 additional proteins that do not bind to a specific DNA sequence but that are known or predicted to interact with chromatin (chromatin-associated). Based on these criteria, we included 283 genes in our TF gene set (Table S2 in File S1).

Our goal was to create genetically flexible, well-characterized libraries of yeast strains carrying inducible alleles of TF and kinase genes for systematic analysis of kinase–TF pathways. We reasoned that potential sources of experimental variability caused by copy-number variation would be minimized by integrating the inducible TF and kinase alleles at a common locus in the genome. We used available Gateway expression vectors to move ORFs into an engineered Gateway destination vector that allows targeted integration of galactose-inducible alleles of genes of interest at the *ho* locus, which is not involved in any biological function in lab haploid or diploid strains (Baganz *et al.* 1997; see *Materials and Methods* for details). Correct integration and galactose-inducible protein expression was confirmed in 129 of 149 kinase/cyclin genes (86% success rate) and 239 of 283 TFs (84%) (Table S1 and Table S2 in File S1). We next looked at the relative abundance of each OE protein in our libraries. To provide a reference for the abundance of overproduced proteins in the TF and kinase libraries, we took a semiquantitative approach and binned each protein into a low, medium, or high abundance category, based on its normalized expression assessed using band intensity measurements from western blots (Table S1 and Table S2 in File S1). Previous work reported a strong correlation between the native abundance of the protein as assessed with a chromosomal TAP tag and its level upon OE (Gelperin *et al.* 2005). In contrast to this finding, for a random selection of 51 proteins we found no correlation between the levels of a protein expressed from the *GAL* promoter and its reported level of expression when TAP-tagged at the endogenous locus (Ghaemmaghami *et al.* 2003) (correlation coefficient *r* = 0.15), suggesting that at least for kinases and TFs, endogenous protein abundance is not related to the level of the same protein when it is overproduced (Figure S1A in File S1).

### Functional characterization of kinase and TF libraries

#### Fitness defects associated with overproduction of kinases and TFs:

**Figure 1:**
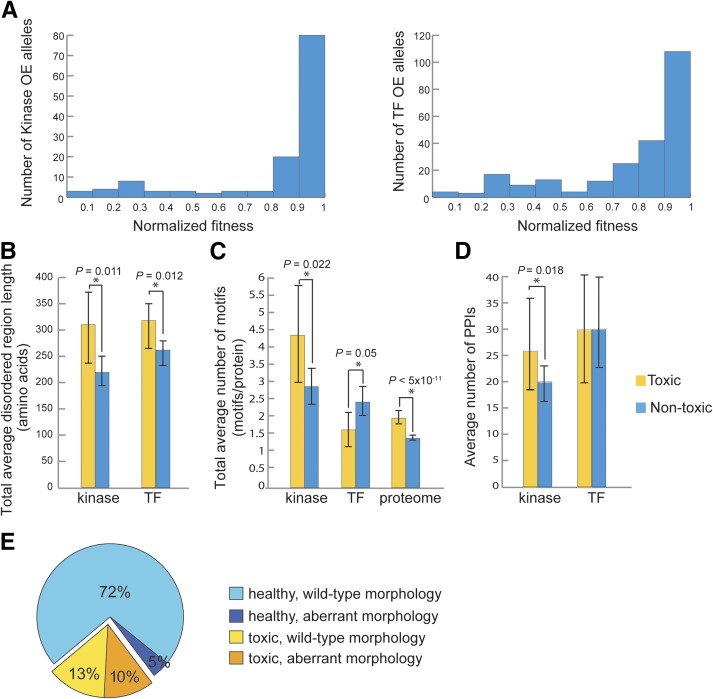
Association of overexpression toxicity of kinases and TFs with growth defects, protein features, and morphology. (A) Cell growth phenotypes caused by kinase or TF overexpression. Bar histograms show the frequency distribution of the relative fitness of strains expressing 129 kinase and 239 TF overexpression alleles upon induction in rich medium containing galactose. The fitness of mutant strains is normalized to a wild-type fitness of one. (B) Analysis of disordered region length in toxic and nontoxic kinases and TFs. Disordered regions of each kinase and TF were obtained from DISOPRED2 and the average lengths of total disordered regions are presented as a bar histogram. Error bars represent minimum and maximum values found in each group. * indicates statistical significance using the Wilcoxon-rank test. (C) Analysis of conserved linear motifs within the disordered regions of kinases, TFs, and the proteome (Sopko *et al.* 2006). The bar histogram shows the average number of linear motifs/protein found in toxic *vs.* nontoxic groups with statistical probability calculated using the Wilcoxon-rank test (* indicates statistical significance). Error bars represent minimum and maximum values found in each group. (D) Analysis of the protein–protein interactions associated with toxic and nontoxic kinases and TFs. The bar histogram shows the average number of protein–protein interactions with statistical probability calculated using the Wilcoxon-rank test. Error bars represent minimum and maximum values found in each group. (E) Summary of morphological phenotypes associated with kinase or TF overexpression. The pie chart summarizes the results of morphology profiling of kinase and TF overexpression strains. OE, overexpression; TF, transcription factor.

One simple measure of the biological consequences of increased gene dosage is the change in growth rate. We measured the average doubling time of the 129 strains comprising the integrated kinase array and the 239 integrated TF strains in OE-inducing conditions (YPGal) and in noninducing conditions (YPD) using an automated spectrophotometer (See *Materials and Methods*). We then calculated the fitness of each strain compared to multiple replicates (∼40) of an isogenic wild-type strain. We used the fitness score to define “toxic” gene sets using an arbitrary cut-off of 0.7 fitness (*i.e.*, growth rate reduced by 30% or more relative to wild-type). This approach identified 26 toxic kinases (20% of kinases tested; Figure 1A and Table S5 in File S1), and 61 toxic TFs (26%; Figure 1A and Table S6 in File S1). Our fitness data overlapped well with other studies that assessed OE phenotypes using plate-based growth assays; 22/26 toxic kinases were previously identified (Table S5 in File S1) and 52/61 overexpressed TFs caused toxicity in other studies (Table S6 in File S1) (Gelperin *et al.* 2005; Sopko *et al.* 2006; Douglas *et al.* 2012). Consistent with previous work (Sopko *et al.* 2006), most toxic kinases in the integrated library were involved in cell cycle progression (*CLB2*, *CLB3*, *CLB6*, and *SWE1*), signaling pathways related to cell growth (*TPK1*, *TPK2*, *TPK3*, and TOR kinases), cell morphogenesis and polarity (*ARK1*, *PRK1*, *AKL1*, *CLA4*, and *GIN4*), or the stress response (HOG pathway: *SSK2* and *SSK22*). (Gelperin *et al.* 2005; Sopko *et al.* 2006; Douglas *et al.* 2012). In total, 85 of the 368 kinases and TFs tested were toxic upon OE (two genes, *TPK1* and *TPK2*, were defined as both kinase and TF). Most of the kinases and TFs that were toxic upon OE had no effect on cell growth when deleted (72/85; Giaever *et al.* 2002), illustrating the potential utility of the OE strains for studying these important regulators in the context of their gain-of-function phenotypes.

To explore the nature of toxicity caused by kinase or TF OE, we examined several inherent features of individual proteins that may correlate with dosage toxicity. First, we asked whether toxicity had any relationship with the abundance of the overproduced protein. We divided all kinases and TFs into three groups by their protein levels upon OE (low, medium, and high; Table S1 and Table S2 in File S1) and found no difference in their average fitness (Figure S1B in File S1). Next, we assessed the total length of disordered regions in kinases and TFs using DISOPRED2 (Ward *et al.* 2004), since the intrinsic disorder content of proteins is a good predictor of dosage toxicity for genes in yeast (Vavouri *et al.* 2009). Indeed, the toxic kinases and TFs had longer total disordered content than their corresponding nontoxic sets, with an average total length of disordered regions for toxic kinases of 309 aa *vs.* 218 aa for nontoxic kinases (Wilcoxon-rank test, *P* = 0.011) and an average total length of 316 aa for disordered regions in toxic TFs *vs.* 257 aa in nontoxic TFs (Wilcoxon-rank test, *P* = 0.012; Figure 1B). We also found a higher average number of evolutionarily conserved short linear motifs (Nguyen Ba *et al.* 2012) in toxic kinases relative to nontoxic kinases (average of 4.4 motifs/kinase *vs.* 2.8 motifs/kinase; Wilcoxon-rank test *P* < 0.05; Figure 1C). This trend was also observed at the proteome level (*P* < 5 × 10^−11^) using a set of toxic proteins described previously (Sopko *et al.* 2006). Toxic kinases had more reported PPIs, consistent with the idea that perturbation of PPIs may cause unexpected phenotypes (average number of PPIs in toxic kinases was 26 *vs.* 20 in nontoxic kinases; Wilcoxon-rank test *P* = 0.018; Figure 1D).

In contrast to kinases, toxic TFs contained a lower average number of conserved motifs in disordered regions than the nontoxic group (average number of conserved motifs for toxic TFs = 1.6 motifs per protein *vs.* nontoxic TFs = 2.4 motifs per protein, Wilcoxon-rank test *P* = 0.05; Figure 1C), and TF toxicity was not related to the number of PPIs attributed to a given TF (average number of PPIs in toxic TFs was 29.9 *vs.* 30.1 in nontoxic TFs; Wilcoxon-rank test *P* = 0.15; Figure 1D). These results suggest that TF OE toxicity is unlikely to be related to promiscuous protein binding, so we tested whether it could be related to promiscuous DNA binding. We used three different measurements as a proxy for the biophysical DNA-binding specificity of a TF: the information content of expert-curated DNA-binding motifs (YeTFaSCo; de Boer and Hughes 2012); the number of predicted sites in the genome using these binding motifs; and the number of synthetic oligonucleotides bound in *in vitro* protein binding microarray (PBM) assays (Badis *et al.* 2008; Zhu *et al.* 2009). In all three tests, we could not find a clear relationship with TF toxicity (Table S7 in File S1). In summary, in contrast to kinases, toxicity of TFs appears unrelated to promiscuous PPIs through short linear motifs and does not appear to be related to promiscuous interactions with DNA based on several parameters that were tested.

#### Characterization of morphological defects associated with kinase and TF OE:

We next developed a cell biological assay to provide a more sensitive phenotypic readout for assessing the consequences of TF or kinase OE. We introduced our TF and kinase OE alleles into strains carrying a GFP reporter gene to mark the cytoplasm (Rps1b-GFP, BY4877, Table S3 in File S1) and an Hta2-mCherry reporter to mark the nucleus. Strains were grown to midlog phase and cell images were collected from three channels (DIC, GFP, and RFP) using fluorescence microscopy, and manually assessed for aberrant phenotypes in 17 subcategories (Table S4 in File S1). Fifty-four of 362 kinase and TF OE strains (15%) displayed morphological defects (Table S8 in File S1), which were often associated with compromised fitness (Figure 1E; 38/54 strains with aberrant morphology were in the toxic set), a dual phenotype that was more likely to be seen as gene toxicity increased (78% of strains with fitness < 0.3 had a clear defect). Many genes (15/38; 39%) whose OE caused both fitness and morphological defects were involved in aspects of cell cycle control and caused obvious nuclear or cell division phenotypes (Table S8 in File S1). In contrast to those with fitness defects, overproduction of some kinases and TFs (*MCK1*, *MKK2*, *FRK1*, *RCK1*, *YRR1*, *RPB3*, *YKL222C*, *WAR1*, *DIG1*, *IOC4*, *MATALPHA1*, and *GAL3*) caused a range of morphological defects with little impact on cell growth. This result affirms the importance of using multiple phenotypic readouts to assess consequences of genetic perturbations (Vizeacoumar *et al.* 2010; Li *et al.* 2011), and confirms the utility of OE alleles in identifying gene function.

### Dosage-dependent GIs between TFs and kinases reveal regulatory relationships

#### SDL interactions:

**Figure 2:**
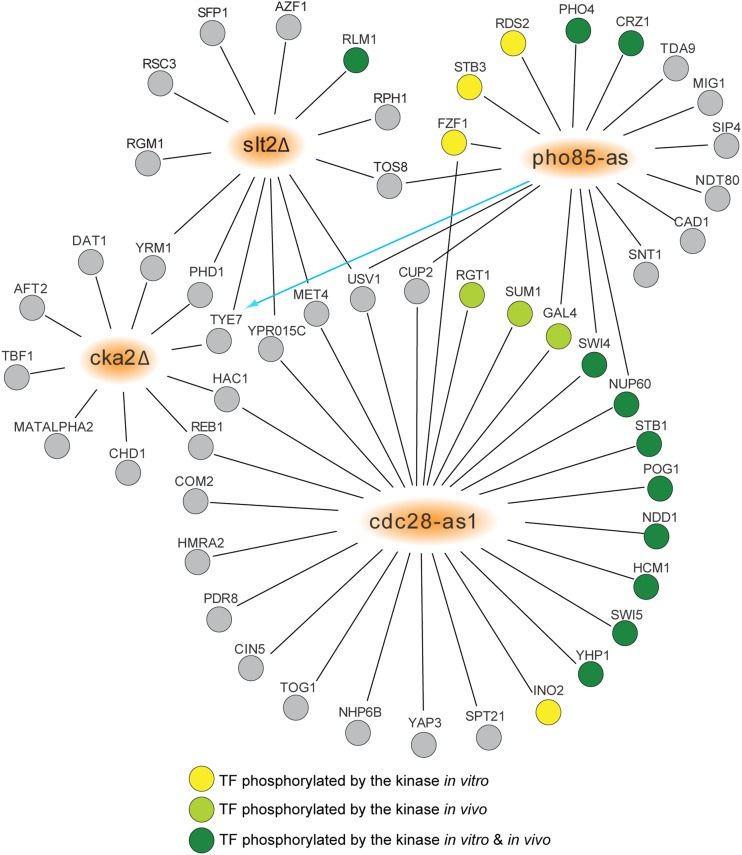
SDL interactions involving loss-of-function kinase mutations and overexpression alleles of TFs. Network diagram showing 68 confirmed GIs between 52 TF overexpression alleles and four loss-of-function kinase alleles (*pho85-as*, *cdc28-as*, *slt2*Δ, *cka2*Δ; orange nodes). TF overexpression alleles are represented as nodes connected to kinase alleles by edges, which indicate either positive (blue line; arrowhead indicating direction of suppression) or negative (black line) GIs. Evidence in the literature showing TF phosphorylation by the interacting kinase is indicated by node color: yellow (*in vitro*); light green (*in vivo*); and dark green (both *in vivo* and *in vitro*). GIs, genetic interactions; SDL, synthetic dosagelethality; TF, transcription factor.

As noted earlier, SDL interactions have been successfully used to discover targets and regulatory pathway components for kinases and other enzymes (Sopko *et al.* 2006; Liu *et al.* 2009; Kaluarachchi Duffy *et al.* 2012; Sharifpoor *et al.* 2012). To validate our integrated TF library, and to expand on previous work, we performed six SDL screens to test GIs between OE alleles of TFs and hypomorphic or LOF alleles of kinases. We chose two CDKs with multiple known substrates that are TFs, the essential cell cycle regulatory CDK *CDC28* and the nonessential multifunctional CDK *PHO85*, and screened them using *as* alleles. The *as* allele allows specific and rapid inhibition of kinase activity in the presence of a chemical inhibitor (Bishop *et al.* 2000; Carroll *et al.* 2001). We also screened deletion alleles of kinases involved in aspects of cell growth through regulation of TF activity and gene expression control: cAMP-dependent protein kinase (PKA; protein kinase A) catalytic subunit *TPK2*, the casein kinase 2 (CK2) catalytic subunits *CKA1* and *CKA2*, and the cell wall integrity MAP kinase *SLT2*. Using the SGA method, we introduced each kinase mutant allele into the stable OE library of TFs (239 TFs) and assessed changes in colony size (fitness) after induction of TF expression to score for positive or negative GIs. GIs were identified by eye, then were individually confirmed by manually regenerating the double mutants using SGA haploid selection and testing their fitness using serial spot dilutions. Frequency of confirmation varied among the kinase mutants tested, ranging from ∼40 to 80%. Confirmed GIs were assessed for other evidence supporting a kinase-substrate relationship by searching public databases [yeast KID (Kinase Interaction Database) and BIOGRID (Biological General Repository for Interaction Datasets), queried April 2016; Sharifpoor *et al.* 2011; Chatr-Aryamontri *et al.* 2015] and the literature. In total, we discovered 68 unique SDL interactions between 52 TF OE alleles and four kinase mutants (*cdc28-as1*, *pho85-as*, *slt2*Δ, *cka2*Δ) (Figure 2 and Table S9 in File S1), 64 of which are novel SDL interactions, expanding the number of dosage lethal GIs between kinases and TFs (∼300) by 20% (Sharifpoor *et al.* 2011).

As noted above, previous work has shown that SDL interactions involving kinases are enriched in kinase-substrate pairs (Sopko *et al.* 2006; Sharifpoor *et al.* 2012). Consistent with these studies, we identified known kinase-substrate pairs in our SDL datasets. Among the 27 TFs that caused a slow growth or lethal phenotype when overproduced in a *cdc28-as1* strain (Figure 2), eight are characterized targets of Cdc28 (Swi4, Nup60, Stb1, Pog1, Ndd1, Hcm1, Swi5, and Yhp1; defined by both *in vitro* and *in vivo* evidence) and four are phosphorylated by Cdc28 either *in vivo* or *in vitro* (Ubersax *et al.* 2003; Holt *et al.* 2009) (∼threefold enrichment; *P*-value < 0.005). Similarly, of the 18 TFs that were specifically detrimental to cell growth when Pho85 activity was inhibited (Figure 2), two are well-known targets of the kinase that were identified in a previous SDL screen with a *pho85*Δ allele, Pho4 and Crz1 (Sopko *et al.* 2006), and three TFs (Rds2, Stb3, Fzf1) are phosphorylated by Pho85 kinase *in vitro* (Dephoure *et al.* 2005). Finally, our SDL screens identified Rlm1, a well-characterized substrate of Slt2 (Dodou and Treisman 1997), along with 12 other TFs whose OE caused a dramatic fitness defect in the *slt2*Δ mutant (Figure 2) and 10 TFs whose OE was toxic in the *cka2*Δ strain. The identification of known targets in our screens validates our integrated TF library as a useful resource for discovering SDL interactions, and suggests that additional substrates may be found among the other TFs that caused synthetic dosage lethality in kinase mutants.

**Figure 3:**
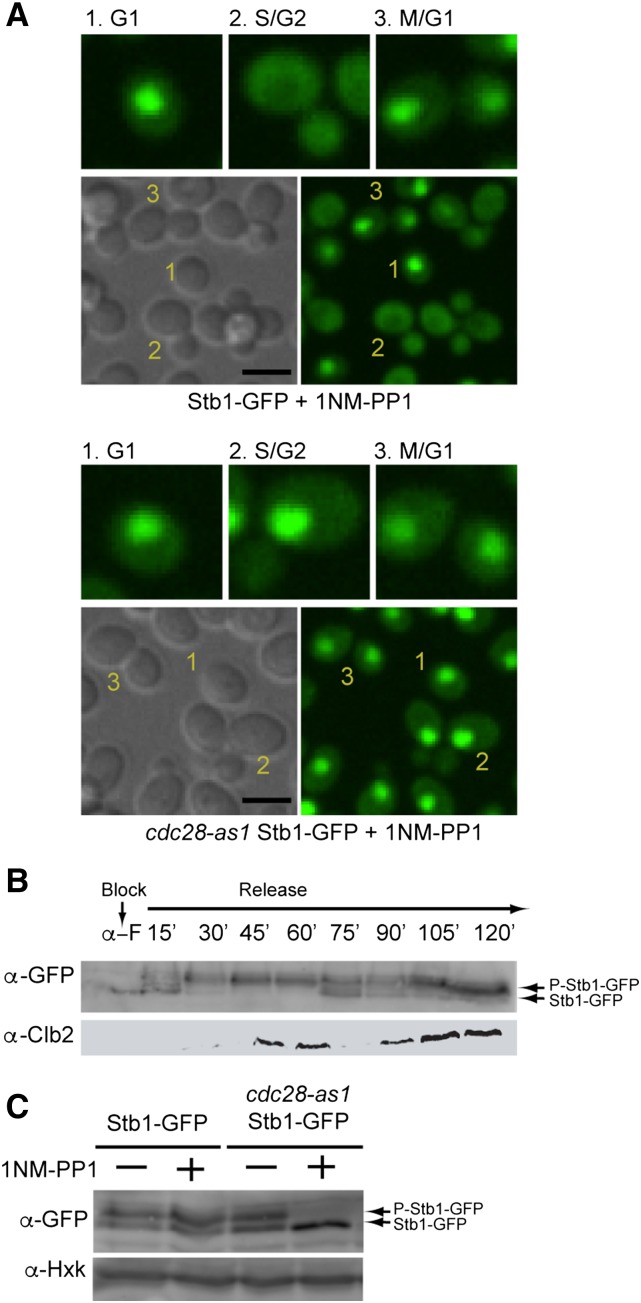
Stb1 localization at different stages of the cell cycle in wildtype and *cdc28-as1* mutant cells. (A) DIC and GFP confocal microscopy images showing wild-type (top panel) and *cdc28-as* cells (bottom panel) expressing C-terminally-tagged Stb1-GFP treated with 5 μM 1NM-PP1 for 40 min. Enlarged images of cells in specific cell cycle stages, as indicated in the large panel below, are shown at the top of each panel: (1) G1 phase; (2) S/G2 phase; and (3) M/G1 phase. Scale bar, 5 μm. (B) Western blot analysis of Stb1-GFP protein extracted from wild-type yeast cells progressing synchronously through the cell cycle following α-factor block and release. Progression through the cell cycle was monitored by western blotting for Clb2 (G2/M; Richardson *et al.* 1992). The arrows indicated the position of migration of Stb1-GFP and phosphorylated Stb1-GFP. (C) Western blot analysis of Stb1-GFP protein extracted from log phase cultures of wild-type and *cdc28-as1* strains treated with carrier or 1NM-PP1 for 40 min. The arrows indicate the position of migration of Stb1 and phosphorylated Stb1. Hxk was used as a loading control (bottom panel). DIC, differential interference contrast; GFP, green fluorescent protein; Hxk, hexokinase.

To explore the potential regulatory relationship behind kinase–TF SDL interactions, we next assessed TF localization changes in the absence of kinase activity. We used SGA to introduce 21 endogenously GFP-tagged versions of the TFs that showed an SDL phenotype into the *cdc28-as1* strain and surveyed changes in the TF-GFP localization or intensity (abundance) after a 40–50 min inhibition of the Cdc28 kinase (Bishop *et al.* 2000) (we tested all TFs showing GIs with *CDC28*, with the exception of six TFs that we could not find in the GFP library: *COM2*, *USV1*, *HMRA2*, *YPR015C*, *TOG1*, and *GAL4*). We discovered two TFs whose localization dramatically changed upon kinase inactivation: Stb1 and Yhp1. Stb1 regulates G1-specific transcription and is phosphorylated by Cln1/Cln2-Cdc28 *in vitro* (Ho *et al.* 1999; Costanzo *et al.* 2003; de Bruin *et al.* 2008). It binds to G1-specific promoters during G1 phase; in *cln1*Δ *cln2*Δ cells Stb1 displays prolonged binding to G1 promoters and G1 transcripts are increased (de Bruin *et al.* 2008), suggesting that Cln1/2-Cdc28 negatively regulates Stb1. We examined Stb1-GFP localization in different cell cycle stages in an asynchronous population. In wild-type cells, Stb1-GFP was nuclear during G2/M and G1 but became cytoplasmic during S/early G2 [∼3–5% of small or medium-budded cells showed a nuclear GFP signal (*n* = 100; Figure 3A)]. This localization change was dependent on Cdc28 kinase activity, as Stb1-GFP stayed in the nucleus throughout S/early G2 upon *cdc28-as1* inhibition [100% of small- and medium-budded cells showed a nuclear GFP signal (*n* = 112); Figure 3A second panel], consistent with the prolonged binding to G1-specific promoters in a *cln1cln2* mutant (de Bruin *et al.* 2008). Stb1 nuclear export in wild-type cells coincided with the appearance of hyperphosphorylated forms of Stb1 (Figure 3B), suggesting that phosphorylation by Cdc28 may promote nuclear exit of Stb1 during S/G2. Supporting this model, Stb1 phosphorylation was reduced in the *cdc28-as1* mutant upon inhibition in lysates prepared from asynchronous cultures (Figure 3C). In summary, these data suggest that phosphorylation of Stb1 by Cdc28 promotes its active export from the nucleus during S/early G2, helping to confine expression of G1 genes to the proper phase of the cell cycle.

*YHP1* encodes a homeobox domain transcriptional repressor that, together with the MADS box TF Mcm1, binds to early cell cycle box (ECB) elements to restrict expression of ECB-regulated genes to M/G1 phase and to hybrid *YOX*/*MCM1*/*FKH* elements in the promoters of some *CLB2* cluster genes to delay transcription until late M phase (Pramila *et al.* 2002). Yhp1 acts in a manner partially redundant with Yox1, to keep its targets repressed. Yhp1 is phosphorylated by Cdc28, an event that is required for its timely degradation during mitosis, indicating that Cdc28 negatively regulates Yhp1 abundance (Landry *et al.* 2014). We followed Yhp1-GFP localization throughout the cell cycle in asynchronous populations and observed that the Yhp1-GFP nuclear signal peaked in cells in S/G2 phase, at a time when Yhp1 targets are repressed; nearly 70% of small-medium budded cells displayed a nuclear GFP signal (*n* = 112). In contrast, consistent with Yhp1 being degraded in mitosis, Yhp1-GFP was no longer nuclear in cells going through M/G1 phases, when M phase and ECB-regulated genes are expressed (Figure S2 in File S1, top left panel). Upon inhibition of Cdc28 kinase activity, Yhp1 nuclear localization was completely abolished; only ∼2% of small-medium budded cells (*n* = 127) showed nuclear fluorescence and the rest of the population showed no fluorescence (Figure S2 in File S1, bottom right panel). In our western blot analysis of a *cdc28-as1* asynchronous population, Yhp1-GFP protein levels were reduced, suggesting that lack of Yhp1 nuclear signal may be due, at least in part, to reduced protein abundance (data not shown). We conclude that Cdc28 kinase activity is required for proper nuclear localization and/or abundance of Yhp1 during S/G2 phase, suggesting a positive regulatory role for Cdc28. We suggest that Cdc28 likely has two distinct cell cycle roles in Yhp1 regulation; it promotes Yhp1 nuclear localization in S/G2 and then negatively regulates Yhp1 protein during M/G1 (Landry *et al.* 2014). This type of cell-cycle specific activation and inhibition has been observed with other Cdc28 substrates such as Swi6 (Sidorova *et al.* 1995) and Hcm1 (Landry *et al.* 2014).

#### DDL interactions:

**Figure 4:**
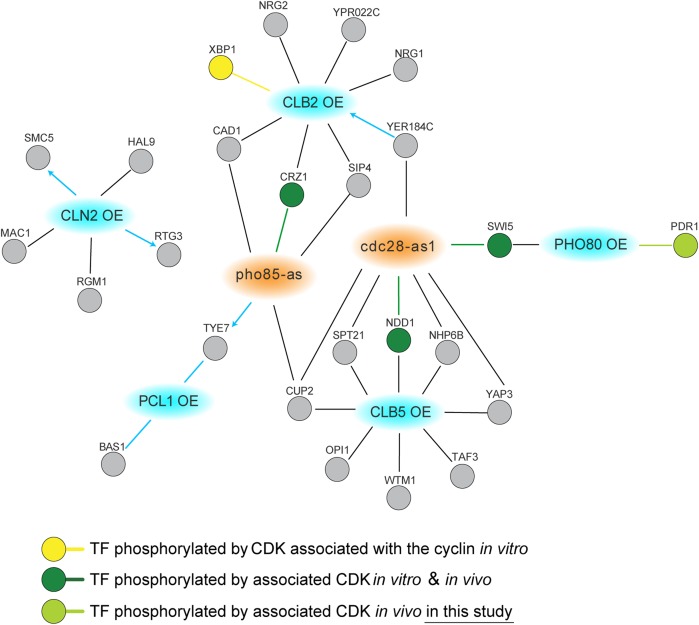
DDL interactions between cyclin and TF OE alleles. Network diagram showing confirmed double dosage interactions between 26 TF overexpression alleles and 5 cyclin OE alleles (blue nodes). Shared synthetic dosage interactions with the corresponding *as* allele of CDKs (orange nodes) are also shown. The TF OE alleles are connected to kinase alleles by edges, which indicate either positive (blue) or negative (black) GIs. For positive interactions, where known, the direction of suppression is indicated with an arrowhead. Evidence from the literature showing TF phosphorylation by the interacting CDK is indicated by node color, yellow (*in vitro*) or dark green (*in vitro* and *in vivo*), and connected to the corresponding CDK or cyclin by a colored edge. The *in vivo* phosphorylation dependence of Pdr1 on Pho80 identified in this study is shown in light green. CDKs, cyclin-dependent kinases; DDL, Double Dosage Lethality; GIs, genetic interactions; OE, overexpression; TF, transcription factor.

Previous systematic SDL screens suggest that one mechanism for SDL involves the failure to properly regulate a substrate in the absence of an upstream kinase or other enzyme (Kaluarachchi Duffy *et al.* 2012; Sharifpoor *et al.* 2012). We reasoned that complementary information might be obtained through cooverproduction of both a regulatory protein and its downstream component. For example, increasing levels of both a kinase and its downstream target may inappropriately activate signaling pathways, leading to a fitness defect. We refer to a GI caused by OE of two genes as DDL. DDL may be particularly useful for discovering positive or agonistic relationships between kinases and their effectors. To expand on the CDK SDL interactions, we chose to test double dosage interactions between cyclins of two CDKs (Cdc28 and Pho85) and TFs. Cyclins are limiting components for activation of CDKs, and cyclin OE is known to hyperactivate CDKs (Wilson *et al.* 1999; Bhaduri and Pryciak 2011). To discover DDL relationships between CDKs and TFs, we introduced GAL-inducible alleles of seven different cyclins (*CLN2*, *CLB5*, *CLB2*, *PCL1*, *PCL2*, *PCL9*, and *PHO80*) on low-copy plasmids (FLEX collection; Hu *et al.* 2007) into the TF OE array and looked for growth changes upon induction of both cyclin and TF expression. Among seven cyclins tested, five cyclins (Cln2, Clb5, Clb2, Pcl1, and Pho80) exhibited DDL phenotypes with 26 unique TFs (21 negative and five positive GIs; see Figure 4 and Table S10 in File S1). Our screen identified two known Cdc28 substrates that had DDL interactions with Cdc28 cyclins: (1) *NDD1*, which had a DDL interaction with *CLB5*, encodes a well-characterized substrate of Cdc28, likely through Clb2 (Reynolds *et al.* 2003); and (2) *XBP1*, which had a DDL interaction with *CLB2*, encodes an *in vitro* substrate of Cdc28 (Ubersax *et al.* 2003; Koivomagi *et al.* 2011).

Pho80-Pho85 regulates responses to environmental and nutritional changes, such as phosphate limitation and stress-induced calcium signaling. OE of *PHO80* gave negative GIs with two TFs: *SWI5* and *PDR1*. Swi5 is a known target of both Cdc28 and Pho85 (Moll *et al.* 1991; Measday *et al.* 2000); it is phosphorylated by Pho85-Pho80 kinase *in vitro* and shows an SDL interaction with *pho85*Δ (Measday *et al.* 2000; Wysocki *et al.* 2006). *PDR1* encodes a zinc cluster TF that controls expression of multidrug resistance genes and plays an important role in the pleiotropic drug response. Similar to *pdr1*Δ, both *pho80*Δ and *pho85*Δ mutants share a broad spectrum of sensitivities to drugs (Huang *et al.* 2002), suggesting that Pho80-Pho85 may function in the same pathway as Pdr1. Consistent with this idea, we found that OE of *PHO80* and *PDR1* both led to significant growth defects in high osmolyte-containing medium (data not shown).

**Figure 5:**
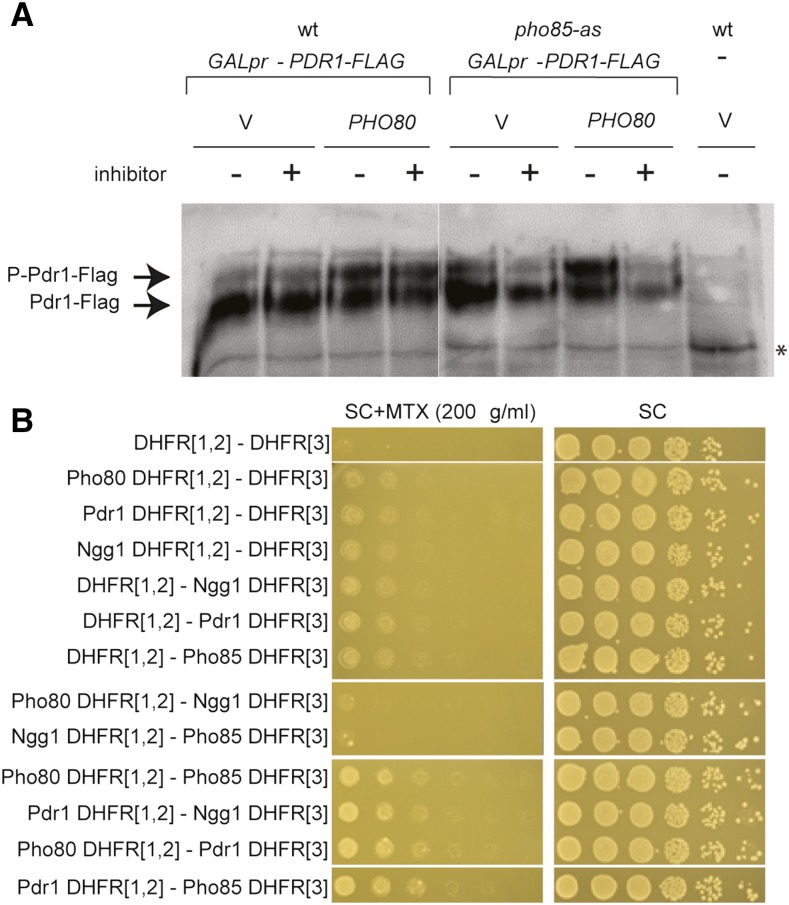
Post-translational modification of Pdr1 depends on *PHO80*. (A) Western blot analysis of Pdr1-FLAG protein cooverexpressed with empty vector or *PHO80* in wt and *pho85-as* strains in the presence and absence of 10 μM 1-Na-PP1. Extracts were prepared from strains grown for 5 hr in YPGal then treated with carrier or inhibitor for 2 hr, and were analyzed on a Phos-tag gel. * indicates nonspecific band reacting with anti-FLAG antibody. (B) Protein–protein interactions measured by DHFR PCA. Cultures of strains expressing the indicated PCA fragments were grown to midlog phase, then serially diluted and spotted on SC and SC + MTX plates. DHFR, dihydrofolate reductase; MTX, methotrexate; PCA, protein-fragments complementation assay; SC, synthetic complete; wt, wild-type; YPGal, rich medium containing 2% galactose.

To see whether any of the TFs discovered in the DDL screen might be a potential substrate for their genetically interacting cyclin–CDK complex, we screened for phosphorylation status changes in TF proteins when the interacting cyclin is coexpressed using Phos-tag SDS polyacrylamide gels for western blotting. We found multiple migrating forms of Pdr1 when its interacting cyclin, *PHO80*, was cooverexpressed (Figure 5A). This post-translational modification was largely dependent on Pho85 kinase activity, as inhibition of *pho85*-as with 1-Na-PP1 abrogated the effect of *PHO80* on Pdr1 protein migration (Figure 5A). Cyclins physically interact with their substrates, and it is the cyclin that confers target specificity of the cyclin–CDK complex (Huang *et al.* 1998; Wilson *et al.* 1999). To test whether Pdr1 binds to Pho80 cyclin or Pho85 CDK, we utilized dihydrofolate reductase (DHFR) PCA. PCA allows detection of physical interactions *in vivo*; when two proteins fused to complementary fragments of a reporter protein interact, the reporter pieces are brought together to restore reporter activity. In this case, the N-terminal fragment DHFR[1,2] and the C-terminal fragment DHFR[3] regenerate DHFR enzyme activity, which allows growth in medium containing methotrexate (Remy and Michnick 1999). In our PCA experiment, we observed growth in the Pdr1-DHFR[1,2] Ngg1-DHFR[3] reporter strain on methotrexate selective medium, confirming a known interaction between Ngg1 (a repressor) and Pdr1, whereas no growth was observed in Pho80-DHFR[1,2] Ngg1-DHFR[3] or Ngg1-DHFR[1,2] Pho85-DHFR[3] reporter strains (Figure 5B). Similarly, we saw a PCA interaction read-out in the Pho80-DHFR[1,2] Pdr1-DHFR[3] strain, as well as the Pdr1-DHFR[1,2] Pho85-DHFR[3] strain. Together, our data suggest that the Pho85-Pho80 complex physically interacts with Pdr1 and promotes its phosphorylation.

## Discussion

Systematic analyses of GIs between LOF alleles of nonessential genes have revealed relationships between functionally diverse pathways and discovered genes that function in the same biological process (Costanzo *et al.* 2010, 2016). However, the frequency of GIs in standard growth conditions is low, probably due to robust genetic buffering relationships (Costanzo *et al.* 2012) and the fact that some biological pathways are inactive in standard growth conditions. GIs involving gain-of-function alleles may address these limitations and provide complementary information (Sharifpoor *et al.* 2012) by allowing facile analysis of essential genes and genes with no clear LOF mutant phenotype (Chua *et al.* 2006; Sopko *et al.* 2006).

In this study, we assembled and characterized high quality collections of yeast strains carrying integrated inducible OE alleles of kinases and TFs. We describe growth defects associated with ∼25% of the OE alleles, a set that consists of 26 toxic kinases and 61 toxic TFs. Consistent with previous work examining OE toxicity at the proteome level (Vavouri *et al.* 2009), we found a significant correlation between kinase toxicity and the number of short linear motifs, which could promote promiscuous PPIs. However, evidence in the literature that clearly addresses the mechanism of OE toxicity for 11 of the 26 toxic kinase and cyclin genes does not support the interaction promiscuity hypothesis (Table S11 in File S1), since toxicity associated with kinase OE can often be suppressed by deletion of downstream effectors (Table S11 in File S1). Specifically, the toxicity associated with OE of four kinases (*SWE1*, *CLA4*, *KIN4*, and *SSK2*), is suppressed by deletion of a known downstream target. For another six kinases, kinase-dead alleles were no longer toxic upon OE (*TPK1*, *GIN4*, *KIN2*, *ARK1*, *PRK1*, and *CDC5*), suggesting that toxicity reflects uncontrolled kinase activity. Finally, for one kinase gene (*TOR1*), OE toxicity appears to result from reduced activity, likely due to disruption of a protein complex, consistent with the balance hypothesis (Papp *et al.* 2003). Direct evidence that promiscuous protein interactions cause toxicity would be difficult to acquire; however, suppression of toxicity by deletion of a downstream substrate or toxicity that depends on enzyme activity suggest that promiscuous PPIs are not the determining factor for the toxic phenotype. In summary, experiments in the literature support increased or unregulated activity (likely at an improper time or place), mediated by linear motifs in the disordered regions, as a mechanism of OE toxicity for many kinases. This suggests that even when a kinase with a high number of linear motifs is overproduced, the linear motifs may retain their specificity and the kinase maintains its original function rather than acquiring a new role through promiscuous interactions.

Our study found that toxic TFs tend to have longer disordered regions, but we did not observe any significant correlation with features known to promote PPIs or DNA-binding specificity. Consistent with our observations on kinases, other evidence suggests that many overexpressed TFs may be toxic because of increased or unregulated activity, rather than promiscuous interactions. A large-scale microarray study found that OE of 55 toxic TFs gave transcriptional changes indicative of increased activity, typically including established targets and genes with consensus promoter motifs, whereas OE of 23 well-characterized TFs that were not toxic produced no significant changes (Chua *et al.* 2006). Thus, as for kinases, evidence from the literature suggests that toxicity of overproduced TFs reflects their original function(s), rather than new roles acquired through promiscuous interactions, suggesting that analysis of the phenotypic consequences or OE of regulatory proteins will provide biologically relevant information.

We probed GIs between kinases and TFs by OE TFs in a variety of kinase mutant backgrounds. The SDL network of *cdk* *as* alleles was enriched for substrates and SDL interactions guided identification of novel regulatory relationships. Among the TFs that had an SDL interaction with *CDC28*, we identified two whose localization or abundance was dependent on Cdc28: Stb1 and Yhp1. Stb1, a positive regulator of late G1 gene expression, is exported from the nucleus in S/G2 in a *CDC28*-dependent manner. OE of *STB1* alone is not toxic (Table S6 in File S1) and similarly, *cdc28-as1* cells were able to grow in the presence of a low level of inhibitor. We suggest that the SDL interaction arises when overproduced Stb1 remains nuclear in S/G2 in a *cdc28* mutant and promotes higher levels of expression of G1 genes. Likewise, the SDL interaction with Yhp1 may arise when overproduced Yhp1 protein is misregulated in the absence of Cdc28. We further expanded on the CDK interaction network using a novel genetic concept, DDL, to identify interactions between the activating cyclins and TFs. DDL screens of cyclins identified known targets of their cognate CDK (Cdc28-Ndd1, Pho85-Swi5). In addition, when we surveyed the DDL interactions using a secondary biochemical assay to detect post-translational modifications altered by cyclin OE, we found that Pdr1 is hyperphosphorylated in a Pho85-dependent manner when Pho80 is cooverexpressed. We propose that DDL screens can identify regulatory relationships between two genes; however, kinase–substrate relationships only explain a fraction of the DDL network and additional investigation is necessary to find what other functional relationships cause this type of GI. As with any dosage lethal interaction, DDL interactions are complex and may reflect many different types of relationships.

Our SDL and DDL screens revealed 65 unique TFs interacting with at least one of the nine kinase LOF or OE alleles. Five TFs (*NDD1*, *YAP3*, *NHP6B*, *CUP2*, and *SPT21*) displayed a DDL interaction with *CLB5* or *CLB2* and also showed an SDL interaction with *cdc28-as1*. One interpretation of these interactions is that perturbation of TF function can be amplified by either a decrease or increase in CDK activity. This may occur when a TF is positively and negatively regulated by the CDK in different conditions. In fact, some CDK substrates require CDK activity for both their activation and inactivation (Mendenhall and Hodge 1998; Enserink and Kolodner 2010). For example, Cdc28 phosphorylates Hcm1 to stimulate DNA binding and phosphorylates Hcm1 (on different residues) to target it for degradation (Landry *et al.* 2014). Another possibility is that Clb2 or Clb5 overproduction has a dominant negative effect on Cdc28 activity and cyclin OE is, thus, mimicking the effects of a LOF mutation in *CDC28*.

In summary, we describe the construction and characterization of libraries of strains carrying integrated wild-type OE alleles of kinases and TFs, and describe two genetic approaches that make use of these libraries to explore gene function and GIs. We show that the TF and kinase collections can be used to interrogate increased gene dosage in the contexts of different genetic backgrounds. We also demonstrate through experimental validation of several interactions that dosage-based interactions can reveal new functional relationships.

## Supplementary Material

Supplemental material is available online at www.g3journal.org/lookup/suppl/doi:10.1534/g3.116.038471/-/DC1.
