# Supplementary material for: Functional Analysis of Kinases and Transcription Factors in Saccharomyces cerevisiae Using an Integrated Overexpression Library
Source: G3 (Bethesda). 2017 Jan 22;7(3):911–21. doi: 10.1534/g3.116.038471 (PMC5345721; doi:10.1534/g3.116.038471)
Supplement: Supplementary file 1 [file 911FigureS1.pdf]

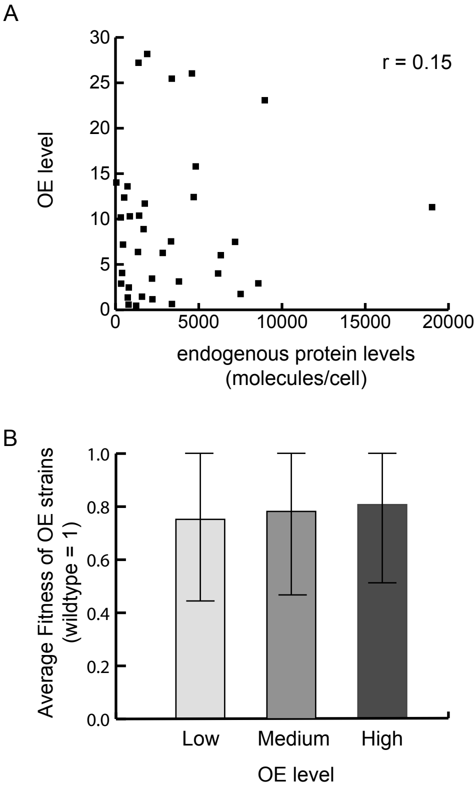

Figure S1. Levels of over-expressed TFs and kinases do not reflect their endogenous protein levels and are not related to fitness phenotypes. A. Scatter plot showing levels of protein overexpression for 51 kinases and transcription factors (y-axis) and their corresponding endogenous protein levels (x-axis). OE protein levels of 51 randomly sampled kinase and TF overexpression strains were assessed by VersaDoc™ and normalized to the level of the background band in α-FLAG western blots (y-axis). Endogenous protein levels of the selected proteins were taken from a previous study of TAP-tagged proteins (Ghaemmaghami et al. 2003; x-axis). The relation between OE and endogenous protein levels is measured using correlation coefficient (r), r= 0.15.

B. Average fitness of strains overexpressing kinase/TF proteins grouped by their OE protein levels. Levels of OE proteins (364 kinase and TF strains) were semi-quantitatively assessed and binned into low, medium and high abundance based on band intensity in western blots. Proteins showing lower band intensity than the α–FLAG background band were binned to low, proteins with similar intensity were binned to medium and proteins with higher than two-fold intensity were binned to high abundance (Supplementary Table 1 and 2). The individual strain fitness was calculated from doubling time (D) observed in liquid growth curve assays: OE fitness = Dwild-type/DOE (Supplementary Table 1 and 2). Error bars indicate the minimum and maximum fitness values in each group.
