# Supplementary material for: Functional Analysis of Kinases and Transcription Factors in Saccharomyces cerevisiae Using an Integrated Overexpression Library
Source: G3 (Bethesda). 2017 Jan 22;7(3):911–21. doi: 10.1534/g3.116.038471 (PMC5345721; doi:10.1534/g3.116.038471)
Supplement: Supplementary file 2 [file 911FigureS2.pdf]

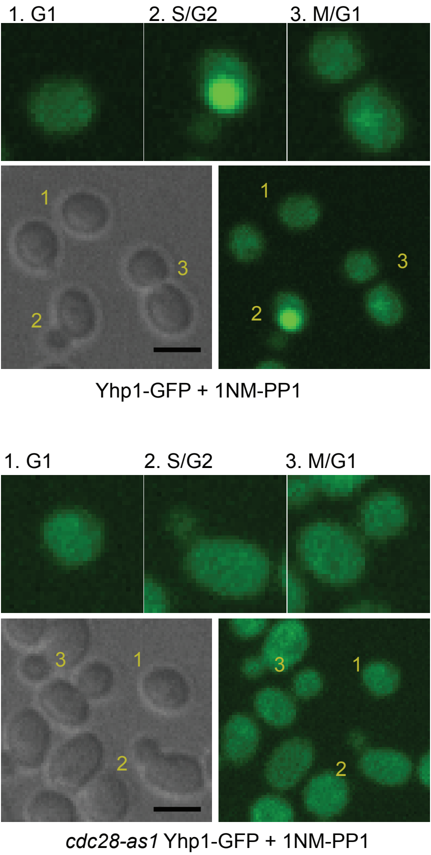

Figure S2. Yhp1 localization at different stages of the cell cycle in wild-type and cdc28-as mutant strains. DIC and GFP confocal microscopy images showing cells from asynchronous cultures of wild-type and cdc28-as1 strains expressing Yhp1-GFP treated with 5μM 1NM-PP1 for 40 minutes. The top three panels show enlarged images of representative cells in specific cell-cycle stages as indicated: 1- G1 phase, 2 – S/G2, 3- M/G1. Scale bar, 5μm.
